# Multi-omic landscape of rheumatoid arthritis: re-evaluation of drug adverse effects

**DOI:** 10.3389/fcell.2014.00059

**Published:** 2014-11-04

**Authors:** Paolo Tieri, XiaoYuan Zhou, Lisha Zhu, Christine Nardini

**Affiliations:** ^1^IAC - Istituto per le Applicazioni del Calcolo “Mauro Picone,” CNR - Consiglio Nazionale delle RicercheRome, Italy; ^2^Group of Clinical Genomic Networks, Key Laboratory of Computational Biology, Chinese Academy of Sciences - Max Planck Society Partner Institute for Computational Biology, Shanghai Institutes for Biological SciencesShanghai, China

**Keywords:** rheumatoid arthritis, multi-*omic* data integration, host-microbiome interface, protein-protein interaction, network topology

## Abstract

**Objective:** To provide a frame to estimate the systemic impact (side/adverse events) of (novel) therapeutic targets by taking into consideration drugs potential on the numerous districts involved in rheumatoid arthritis (RA) from the inflammatory and immune response to the gut-intestinal (GI) microbiome.

**Methods:** We curated the collection of molecules from high-throughput screens of diverse (multi-*omic*) biochemical origin, experimentally associated to RA. Starting from such collection we generated RA-related protein-protein interaction (PPI) networks (*interactomes)* based on experimental PPI data. Pharmacological treatment simulation, topological and functional analyses were further run to gain insight into the proteins most affected by therapy and by multi-*omic* modeling.

**Results:** Simulation on the administration of MTX results in the activation of expected (apoptosis) and adverse (nitrogenous metabolism alteration) effects. Growth factor receptor-bound protein 2 (GRB2) and Interleukin-1 Receptor Associated Kinase-4 (IRAK4, already an RA target) emerge as relevant nodes. The former controls the activation of inflammatory, proliferative and degenerative pathways in host and pathogens. The latter controls immune alterations and blocks innate response to pathogens.

**Conclusions:** This multi-omic map properly recollects in a single analytical picture known, yet complex, information like the adverse/side effects of MTX, and provides a reliable platform for *in silico* hypothesis testing or recommendation on novel therapies. These results can support the development of RA translational research in the design of validation experiments and clinical trials, as such we identify GRB2 as a robust potential new target for RA for its ability to control both synovial degeneracy and dysbiosis, and, conversely, warn on the usage of IRAK4-inhibitors recently promoted, as this involves potential adverse effects in the form of impaired innate response to pathogens.

## Introduction

Rheumatoid arthritis (RA) is a multifaceted autoimmune, chronic and inflammatory disease with, to date, unclear etiology. As a consequence of its complexity, the definition of efficient and effective therapies remains a remarkable challenge due to the difficulties in controlling side effects and adverse events in relation to known (like genetic susceptibility, Stahl et al., 2010) and emergent (epigenomic factors, Nakano et al., 2012, dysbiosis, Scher and Abramson, 2011) RA-associated con-causes.

Recently, translational research has welcomed into medicine a number of novel perspectives. Among these, sequencing technologies (*omic* screens) and computational intensive approaches (systems biology) now coagulate into a practice where technology and mathematical modeling serve basic research in the production of selected hypotheses, which testing *in vitro, in vivo* and ultimately in clinical studies can support medical research and practice (Okada et al., 2014; You et al., 2014). The recent acknowledgment of the importance and complexity of the gut intestinal (GI) microbiome in the onset, progression and regression of RA (Scher and Abramson, 2011; Scher et al., 2012, 2013) and other autoimmune diseases, requires to incorporate the effects on the GI microbiome for any novel therapy. While protocols and medical best practice recommendations become mature in this direction, we propose the use of network approaches and *omics* from diverse origins (i.e., different biochemical districts/compartments/layers) including genomics, epigenomics, transcriptomics, post-transcriptomics, proteomics, and host-microbiome interface to GI metagenomics, to appropriately monitor the complexity of the disease. The novelty of the present work, therefore, lies not only in its application to RA, but also in the number of *omic* layers we have used, from genomic to proteomic and including the host-microbiome interface. These novelties allow to draw a single analytical picture of the fragmented molecular information available to date on RA, an easily consultable and extendable reference map for the researchers in the field, and—importantly—a systemic evaluation on the impact of a recently proposed RA therapeutic target (IRAK4), valuable *per se* and as an exemplar application of this approach. Overall, this work contributes to the general debate about data integration by offering details on our methodology, and to the area of complex inflammatory diseases, by providing specific examples of data choice and operational results.

## Methods

### Map construction

The datasets used to construct the map are gathered from 13 different sources from databases and literature (Table 1). We included molecules experimentally associated to RA from manual curation of literature sources (*core* dataset, *CD*, 377 proteins, Data Sheet 1, Tables S1–S6), and additional molecules and pathways strongly yet not explicitly associated to RA (*extended* dataset, *ED*, 4709 proteins, Data Sheet 1, Tables S3A–E, S7–S13). A summary of all datasets and proteins' Uniprot IDs is provided in Data Sheet 1, Table S14. While the *core* set constitutes a more specific RA map, its extension offers a more systemic and practically usable map, notably in terms of the significance of the statistics that can be run on the extended map. The map presented here assembles genomic, epigenomic, transcriptomic, post-transcriptomic, proteomic, and host-microbiome interface data related to RA, as detailed below, and integrates such information at the functional level of protein-protein interactions (PPIs). The PPI framework is an assessed integrative approach (Hodgman, 2007; Dittrich et al., 2008; Jin et al., 2008; Kim et al., 2010; Iskar et al., 2012) that has already been used in computational biology to understand diseases' pathogenesis (Huang et al., 2009b); to implement tools for the interpretation of inferred gene and protein lists (Berger et al., 2007; Antonov et al., 2009); to prioritize cancer-associated genes (Wu et al., 2012); to predict functional linkages among genes (Lehner and Lee, 2008); to show the implication of protein networks topology in genetics, personal genomics, and therapy (Lee et al., 2013); to implement data integration workflows showcased in obstructive nephropathy in children (Moulos et al., 2011).

**Table 1:** **Data sources, subsets and number of elements of the RA map**.

**Subset Id**.	**Source of subset**	**Main dataset destination**	**No. of proteins in subset**	**Total no. of proteins in main dataset**	**No. of proteins (and PPIs) in the interactome map**	**No. of proteins (and PPIs) in the interactome map: main cluster**
1	GWAS	*Core*	223	377	303 (597)	161 (542)
2	UNIPROT	*Core*	49			
3	Literature review	*Core*	53			
4	Methylation	*Core*	37			
5	Exp. valid. micriob. interface	*Core*	54			
6	NF-κB consensus	*Core*	16			
3A	T cell activation pathways	*Extended*	1248	4709	3783 (24457)	3466 (24364)
3B	Other pathways	*Extended*	283			
3C	Cytokines	*Extended*	1536			
3D	Growth and differentiation	*Extended*	472			
3E	Intracell signaling and TFs	*Extended*	1837			
7	Transcriptional RA map	*Extended*	212			
8	RA-miRNA reg. proteins	*Extended*	1652			
9A	Downreg. genes in RA	*Extended*	451			
9B	Upreg. genes in RA	*Extended*	210			
10	Inflammasomes	*Extended*	152			
11	Adenosine receptors	*Extended*	569			
12	GPCRs	*Extended*	364			
13	Microbiome interface	*Extended*	171			

### Core dataset

The CD is composed of 377 proteins retrieved from six data sources (Data Sheet 1, Tables S1–S6):

1) RA genome-wide association studies (GWAS) gathered and integrated from five different databases (BioGPS (Wu et al., 2009), HuGE (Yu et al., 2008), NHGRI, OMIM, PharmGKB (Klein et al., 2001); see Data Sheet 1, Table S1 for the specific query processes);
2) RA-associated proteins from the Universal Protein Resource (Uniprot) (Consortium, 2010), retrieved using as search parameters “*rheumatoid arthritis*” and “*human*” and then manually screened (Data Sheet 1, Table S2);
3) Genes and proteins retrieved from a comprehensive review of the literature, in particular genes appearing in Tables 1, 2 of Review (Mcinnes and Schett, 2011) and cited references (Data Sheet 1, Table S3);
4) Genes that show epigenetic changes in relation to RA, as specified in Trenkmann et al. (2010); Karouzakis et al. (2011) (Data Sheet 1, Table S4);
5) Proteins that are at the interface between the host and the oral microbiome, in particular proteins experimentally known to be differentially expressed in presence of *Porphyromonas Gingivalis* (Zhou and Amar, 2006), a periodontitis-causing bacterium that has been strongly linked to the insurgence of RA (Mikuls et al., 2012; Scher et al., 2012; Smit et al., 2012; Bingham and Moni, 2013; Ogrendik, 2013; Okada et al., 2013) (Data Sheet 1, Table S5);
6) The key elements of the NF-κB system, the master regulator of inflammation (Oeckinghaus et al., 2011; Smale, 2011; Hayden and Ghosh, 2012) at the center of a complex regulatory interactome (Tieri et al., 2012) prominently implicated in the onset and development of RA (Miagkov et al., 1998; Makarov, 2001; Feldmann et al., 2002; Okamoto, 2006; Roman-Blas and Jimenez, 2006, 2008; Simmonds and Foxwell, 2008; Van Loo and Beyaert, 2011): we included 16 “consensus” proteins that appear at the intersection of the three main NF-κB-related datasets described in Tieri et al. (2012) (Data Sheet 1, Table S6).

**Table 2:**
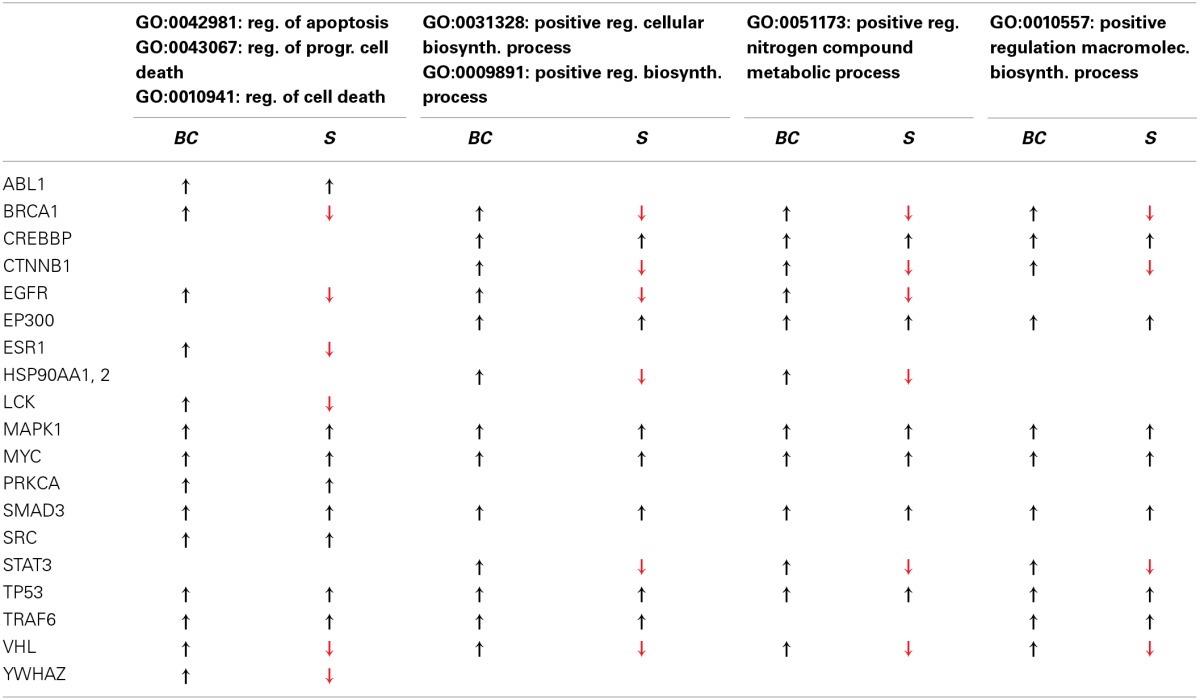
**RA-associated proteins significantly modified upon MTX therapy release and functional annotation clustering in DAVID**.

Thirty-two proteins were identified to be significantly changed by the 20 MTX target proteins' deletion (1000 permutations, adjusted p-value = 0.01). The topological measures of betweenness and stress centrality were shown to be significantly altered increased (black arrow **↑**) or decreased (red arrow 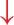) after knocking out the MTX target proteins. Among the listed proteins, enriched for the shown GO categories, STAT3 was found to belong to the host-microbiome interface as defined in Methods. The top 2 functional annotation clusters run on the changed proteins identified enrichment for cell death and biosynthetic process as well as nitrogen compound metabolic process (Functional Annotation Clustering Classification stringency: high, see Supplementary Data Sheet 2, Table S18; BC, betweenness centrality; S, stress centrality).

### Extended dataset

The *extended* dataset (ED, that includes CD) is composed of 4709 proteins, which are involved in a broader sense in the onset and development of RA, such as proteins participating in signaling pathways or cascades of recognized importance for RA. This extension provides a more general setting for the molecular framing of RA, and offers a larger network to operate on, with more relevant statistics and analyses, giving account for contributions coming from entities that may have been neglected or that are not experimentally related to RA, but that participate to the inception of the disease. In addition to the proteins of the *core* dataset, we added eight main subsets, as follows (Data Sheet 1, Tables S3A–E, S7–S13):

3A-B-C-D-E) in retrieving data from Mcinnes and Schett (2011) and references cited there, we considered that some of the key proteins can be “hidden” inside the signaling pathways involved in the disease. In order to take into account such potentially important and usually neglected elements, we expanded subset 3 of CD by a pathway enrichment analysis process, using the genes listed in Mcinnes and Schett (2011) Tables 1, 2. To populate these five subsets, the selected genes have been input in the *pathway over-representation analysis* (ORA) tool of InnateDB, one of the most comprehensive sources of pathways data available (Lynn et al., 2008; Breuer et al., 2013). Pathway ORA has been performed on InnateDB using hypergeometric distribution for *p*-value computation and Benjamini–Hochberg correction method for multiple hypothesis testing. All the proteins participating to such over-represented pathways were then included. We retrieved respectively: 39 enriched pathways accounting for 1248 proteins (subset 3A), 14 pathways and 283 proteins (3B), 46 pathways and 1536 proteins (3C), 5 pathways and 472 proteins (3D), and 92 pathways and 1837 proteins (3E), all collected in Data Sheet 1, Tables S3A–E;
7) Genes derived from the transcriptional RA map in Wu et al. (2010) (Data Sheet 1, Table S7);
8) RA-related miRNA-regulated genes: experimentally validated target genes of all miRNAs that are associated to RA in the database miRWalk (Dweep et al., 2011) (search mode: *holistic view of validated disease-miRNA interactions*; web reference: http://www.umm.uni-heidelberg.de/apps/zmf/mirwalk/disease.html; query keywords: *Arthritis* AND *Rheumatic diseases*) (Data Sheet 1, Table S8);
9A,B) gene expression profiles of RA patients and healthy controls were searched on Gene Expression Omnibus (GEO, (Barrett et al., 2011) http://www.ncbi.nlm.nih.gov/geo/) with the query [“rheumatoid arthritis” AND “(synovi^*^ OR blood)”] (i.e., in synovial tissue and/or blood). In order to include only highly consistent information, datasets without pre-treatment samples, with no details about the therapy and no raw data were filtered out. Human PBMCs collected and processed by Affymetrix technology were selected, leaving only one dataset out of the initial 61, GSE7524, which contains transcriptomic profiles of 2 healthy controls, 2 before and 2 after anti-TNFα treatment samples. Affymetrix Human Genome U133A Array was used to measure the expression levels of ~14,500 well-characterized human genes. The raw data were pre-processed using *affy* package (Gautier et al., 2004) in R (http://www.r-project.org/), normalized using robust multi-array average (*rma*) (Irizarry et al., 2003) and for multiple probes corresponding to the same gene, the probe with the highest standard variation across all samples was used to represent the gene. Differentially expressed genes [fold-change (Murie et al., 2009) =2] were identified with the comparison between the 2 healthy controls and the 2 before anti-TNFα treatment samples resulting in 646 genes differentially expressed, among which 440 genes (451 proteins) were down-regulated and 206 genes (210 proteins) were up-regulated (Data Sheet 1, Tables S9A,B);
10) Proteins related to the inflammasome, a multiprotein oligomer responsible for activation of inflammatory processes proteins, which is also known to be activated from the bacterium *P. Gingivalis*, among others, and recognized to play a relevant role in RA (Sidiropoulos et al., 2008; Kolly et al., 2010; Farquharson et al., 2012; Mathews et al., 2013) (Data Sheet 1, Table S10). This set was retrieved using *ORA* as described in 3A-B-C-D-E;
11) Adenosine receptors and related proteins, known to be involved in RA (Varani et al., 2010, 2011; Vincenzi et al., 2013) and possibly at the basis of the mechanism of action of methotrexate, first-line therapy for the treatment of RA (Stamp et al., 2012) (Data Sheet 1, Table S11). This set was retrieved using *ORA* as in 3A-B-C-D-E and 10;
12) The large family of G Protein Coupled Receptors (GPCRs) (Hutchings et al., 2010; Lozupone et al., 2012; Maynard et al., 2012; Tremaroli and Backhed, 2012), pertaining to host-microbiome interface proteins (grouped in a separate set from 13 due to their numerosity), retrieved from http://www.iuphar-db.org/DATABASE/ReceptorFamiliesForward?type=GPCR (Sharman et al., 2013) (Data Sheet 1, Table S12);
13) The set of host-microbiome interacting proteins, manually curated from recent reviews (Lozupone et al., 2012; Maynard et al., 2012; Tremaroli and Backhed, 2012), to describe the bridge between innate immunity (altered in RA) and the GI microbiome [known to be involved in immune diseases in general and in RA in particular (Scher and Abramson, 2011)]. Globally this dataset accounts for the Toll-like Receptor family (TLRs), the mucin proteins family, selected Immunoglobulins (Ig) and their receptors, among others (Data Sheet 1, Table S13).

Datasets are integrated at the PPI level as peers to avoid introducing any bias *a priori* in the network construction and to warrant that these data are connected in a biologically meaningful way. Protein-protein interactions were retrieved in Cytoscape from the Agile Protein Interaction DataAnalyzer database (APID, Prieto and De Las Rivas, 2006) that includes all known experimentally validated protein-protein interactions from BIND, BioGRID, DIP, HPRD, IntAct and MINT databases, accessed via the APID2NET (Hernandez-Toro et al., 2007) plugin. This process lead to the definitions of, respectively, the core interactome (*CI*, 303 proteins, 597 interactions, high resolution Image S1) and the extended interactome (*EI*, 3783 proteins, 24457 interactions, high resolution Image S2). Discussion on caveats and choices of original sources can be found in Tieri and Nardini (2013).

### Topological analysis

Topological analysis was run separately on the main *connected component* of each interactome (i.e., excluding the proteins for which no PPI was retrieved, i.e., that remained isolated) to evaluate a number of network parameters (Assenov et al., 2008): *degree*, or *connectivity*, i.e., the number of nodes linked to the node of interest (number of edges); and *betweenness centrality* (BC), a measure of the amount of control that a node exerts over the interactions of other nodes in the network. This measure favors nodes that join communities such as dense subnetworks, rather than nodes that lie inside a community, and has been shown to characterize essential proteins (Platzer et al., 2007). All calculated network parameters and rankings are listed in Data Sheet 2, Tables S15, S16 or can be recalculated from the Cytoscape CI_EI.cys (Data Sheet 3) file available at http://www.picb.ac.cn/ClinicalGenomicNTW/RAmultiomic.html.

### Pharmacological treatment simulation

To simulate the pharmacological treatment, a virtual node knockout experiment has been performed by controlling (manual removal of the nodes and Cytoscape plugin *Interference* (Scardoni et al., 2014) 20 MTX controlled targets identified in literature (Cutolo et al., 2001; Chan and Cronstein, 2002) present in EI (Data Sheet 2, Table S17). Betweenness centrality (and, to add robustness to the analysis, stress, S, i.e., an alternative centrality functional form) were then re-calculated to assess the impact of such therapy on the topology and hence the functionality of the network. Manual node removal and pharmacological simulation plugin present overlapping results (*betweenness*: 95.9%, *stress*: 98.2%, Data Sheet 2, Table S17). The *p*-values, corrected for multiple testing (threshold 0.05), have been calculated after constructing null betweenness centrality distributions by 1000 random deletions of 20 nodes, as many as the MTX targets (Efron and Tibshirani, 1993). Functional clustering analysis has been then performed (Data Sheet 2, Table S18).

### Comparative analysis

We further run a comparative analysis between our newly constructed multi-*omic* map, EI, and TR, that represent an earlier transcriptional-only version (Wu et al., 2010), to highlight the biological mechanisms that have been better emphasized from the usage of multilayer *omic* data.

*Degree* was evaluated as the number of edges attached to a node for the undirected networks as EI (and CI) are (i.e., connections among nodes do not indicate *directional* cause-effect nor temporal relationship). For TR (directed network) proteins and their modified instances (such as MAPKs and phosphorylated-MAPKs) were first considered as one (complex) node, then in-degrees (edges *to* the node) and out-degrees (edges *from* the node) of the components (MAPK and phosphorylated-MAPK) were summed up to obtain the undirected degree, after subtracting the number of edges connecting the members of the complex node. To complete the compatibility of the degree defined for undirected maps (and namely EI), given the different sizes of EI and TR, the percentrank of the degree was also computed. The nodes which degree rank was modified by more than 10% between the two networks, were considered as nodes undergoing a *transition*. A node was defined as *accomplished* when its % rank degree was preserved, *loser* when the ranking reduced from TR to EI, *climber* when it increased from TR to EI (Data Sheet 2, Table S19). From a strictly topological point of view, the threshold that defines a node as *accomplished* can be set to zero, and hence this definition identifies only the nodes with the same exact degree. From a biological standpoint, and for an informative biological interpretation of the results, it is not necessary to impose the exact matching of the ranking. For this reason we relaxed the threshold and defined as accomplished the nodes that present the same, higher or lower % rank of the degree with ±10% tolerance, as a reasonable compromise.

Biological meaning for *climbers* and *accomplished* nodes in the transition TR to EI was assessed by enrichment analysis Enrichr (Chen et al., 2013) see Data Sheet 2, Table S20.

## Results and discussion

After curating all molecular information (Table 1) we inferred the network from the reconstructed lists with the PPI approach, which consists of connecting nodes (molecules) based on their interactions at the protein level, a broadly assessed approach in computational biology, and already used for RA in both already cited (Okada et al., 2014; You et al., 2014). All following results pertain to the analysis on the extended interactome (EI), more informative for its larger size.

**Figure 1:**
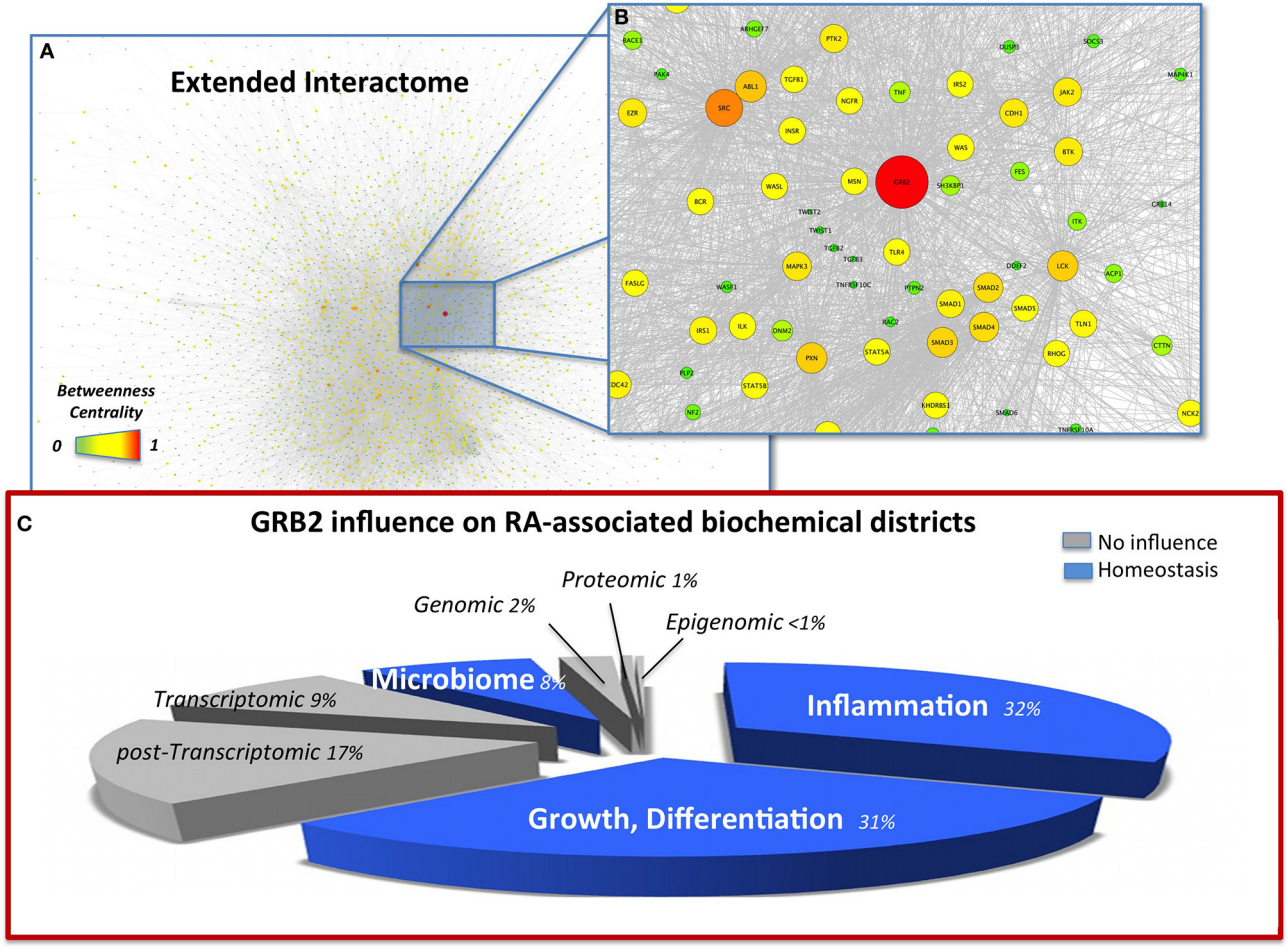
**(A)** Snapshot of the extended interactome (EI) with nodes highlighted by betweenness centrality (BC), high resolution browsable figure provided in Supplementary Files (Image S2). **(B)** Zoom on the top ranking BC node (GRB2) and its closer interactome. Pathways relevant in the indication of GRB2 as an RA target, able to control inflammation TGF-β (TGFB1-3), TNF-α (TNF, TNFRS10C), MAPK (MAP4K1, MAPK3), degeneracy EMT (TWIST1-2, CDH1), and dysbiosis (TRL4) are also highlighted. **(C)** Visual summary of the influence of GRB2 on the RA-affected districts highlight a homeostatic (blue) influence on inflammation, GI microbiome, growth, differentiation. The pie-chart slices' size is proportional to the number of molecules considered in each district. Districts were merged from the total 13 datasets according to biochemical homogeneity in the following 8 categories: Genomic (DNA, Dataset 1); Epigenomic (mDNA, Dataset 4); Transcriptomic (mRNA, Datasets 7, 9A, 9B); Post-transcriptomic (miRNA, Dataset 8); Proteomic (proteins, Dataset 2); Microbiome (Host-microbiome proteins interface, Oral microbiome Datasets 5, 10, 12, 13); Inflammation (6, 3A, 3B, 3C); Others, i.e., Growth, Differentiation (Datasets 3, 11, 3D, 3E).

To validate the ability of our network to model the biomolecular aspects of RA, we first simulated a therapeutic approach with MTX (see methods) and compared the results with the major known effects reported in literature (Figure 1A). As a result of the control on 20 MTX targets removal, the network changes its topology (Figure 1B; Data Sheet 2, Table S17), and the functional analysis indicates that 32 molecules which BC significantly altered (Data Sheet 2, Table S17, col. 2) pertain to two main functions [Data Sheet 2, Table S18, DAVID (Huang et al., 2009a)]: regulation of programmed cell death, a known effect of MTX (Spurlock et al., 2011); and metabolic and biosynthetic processes, an alteration known to constitute a side effect of the treatment (Phillips et al., 2003), as well as an area of synergy between host and microbiome (Tremaroli and Backhed, 2012; Devaraj et al., 2013; Winter et al., 2013). Moving down to the gene level, as illustrated in Table 2, Signal Transducers and Activators of Transcription 3 (STAT3) deserves particular attention, as it is a crucial player in the JAK/STAT signaling cascade, at the basis of the signal transduction mechanism for many cytokine receptors, highly activated in RA (Paunovic et al., 2008), and an important member of the host-microbiome interface (Zhou and Amar, 2006), being involved in the host susceptibility/defense against intestinal infections at the mucosal level (Miettinen et al., 2000).

From a topological point of view, STAT3 presents enhanced *betweenness* and reduced *stress centralities* after virtual MTX treatment. This is an unusual topological condition—since there is commonly correlation between stress and betweenness—where, upon perturbation (MTX) a higher fraction of shortest paths converges on STAT3 (gain in *betweenness centrality*) despite a decrease in their absolute number (loss of *stress centrality*). This indicates that the networks shrinks and STAT3 becomes more important, a fact that can be translated in biological terms as the compensatory mechanisms induced by the loss of some molecules' presence/activity (MTX targets), which globally force STAT3 to become the molecule through which more numerous (higher *betweenness*) but less efficient molecular reactions (longer paths, lower *stress*) occur.

Overall, STAT3, which is already considered a crucial target in RA for its critical role in the T regulatory/helper 17 lymphoid cells [T_reg_/Th_17_ balance overabundant in RA (Leipe et al., 2010)] is coherently shown as an indirectly controlled target by MTX explaining the ability of the therapy to rebalance Th17/IL17 ratio (Li et al., 2012).

In conclusion, our map is able to recollect known and yet complex information about the effects of MTX, this represents an important validation of our frame for further simulations. Additionally, our map indicates a clear link between MTX and dysbiosis, which to date has not been explicitly unrevealed, although enterocolitis is a known toxic effect of MTX, linked to the induced nitroxidative stress (Kolli et al., 2008, 2013). This is a critical fact as the known adverse effects of MTX, generally described as immunodepressive, appear to be composed not only by the known oxidative organ stress, but also by an added dysbiosis, possibly mediated by an overload on STAT3.

The topological analysis highlights the striking relevance of Growth factor receptor-bound protein 2 (GRB2) with values of *BC* more than two-fold (Data Sheet 2, Table S16) compared to the second in rank, the Epidermal growth factor receptor (EGFR). Based on literature, GRB2 is an effective target (Phase I clinical trial, http://www.biopathholdings.com/) for Acute Myeloid Leukemia (AML), Chronic myelogenous leukemia (CML) and Myelodysplastic syndromes (MDS); an important mediator of the oncogenic activities of TGF-β, via epithelial mesenchymal transition (EMT) (Galliher-Beckley and Schiemann, 2008); a crucial player in the host-microbiome interaction of *Helicobacter pylori*, able to induce host cell scattering and proliferation via the activation of the Ras/MEK/ERK pathway (Mimuro et al., 2002); a marker of RA in synoviocites (Huh et al., 2003). GRB2 is additionally activated by leptin (Pai et al., 2005), abundant in RA (Bokarewa et al., 2003) and able to increase *Prevotella intermedia* LPS-induced TNF-α production (Kim, 2010). Moreover, another member of the *Prevotella* genus (*P. copri*) has recently been liaised to RA (Scher et al., 2013), as a specific marker of GI microbiome dysbiosis associated to the disease. When observed from the network perspective this apparently scattered information fits in a connected map (Figure 1B) and hence builds a robust rationale for considering GRB2 as a target for RA. The activation of proliferative and inflammatory pathways as well as EMT, are hallmarks of RA (You et al., 2014) suggesting that the control on GRB2 as a regulator of such mechanisms is appropriate. Additionally, the control on GRB2 exerted by *H. pylori* [already proposed in relation to RA (Melby et al., 1999)] and by *P. intermedia* in the presence of leptin indicate that targeting of GRB2 is not only of relevance to control the phenotypic symptoms of RA (joints degeneracy) but also the recently highlighted dysbiosis that accompany the disease, via the control of the disruptive mechanisms by which pathogens can exert their action on the host (Figure 1C).

**Figure 2:**
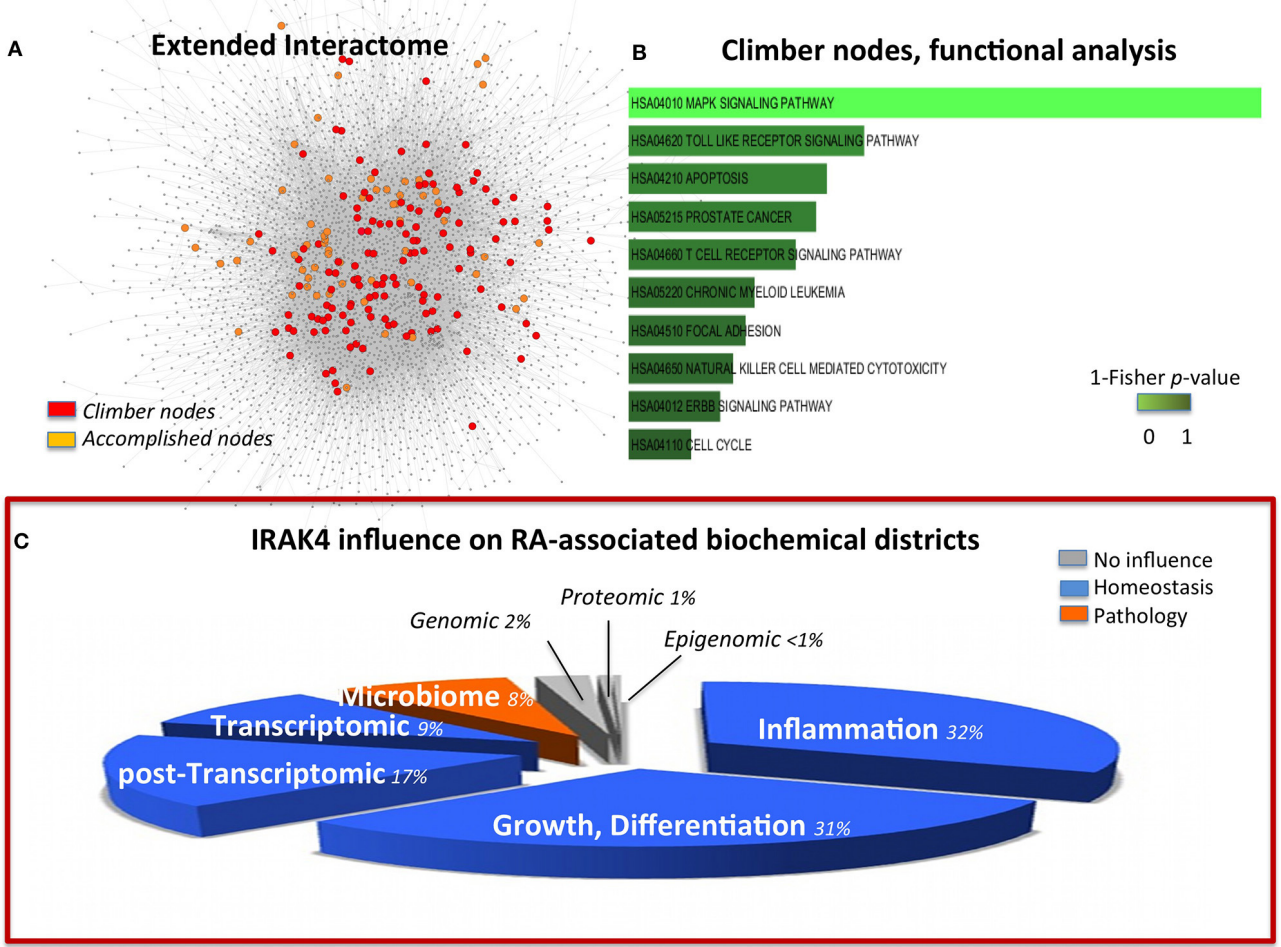
**(A)** Multi-*omic* map (EI) nodes highlighted according to their role in comparison with a transcriptional-only map (TR). In orange, nodes that maintain their role and importance in both EI and TR (*accomplished*); in red, nodes that gain importance in the multi-*omic* context, (*climbers*). **(B)** Functional analysis of the climber hubs, which highlight the striking significance of MAPK signals. Panel **(C)** is built in the same way of Figure 1C to permit easy comparison of the two targets. It represents the summary of the influence of IRAK4 on the RA-affected districts, and highlights a homeostatic (blue) influence on inflammation, growth, differentiation as well as transcriptomic and post-transcriptomic districts. However, the microbiome interface response is impaired by IRAK4 inhibition of the innate immune response to pathogens. The pie-chart slices' size is proportional to the number of molecules considered in each district (as in Figure 1).

Given the relevance of RA as a paradigmatic autoimmune disease, a variety of *in silico* modeling approaches have been devised (Okada et al., 2014; You et al., 2014), and, among those, an early transcriptional only map (hereinafter TR, 302 nodes; Wu et al., 2010). The previous compilation of this simplified version put us in the relatively unique position to be able to quantify the benefit, in terms of information content, of expanding from transcriptional to multi-omic the network modeling of RA. The molecules that gain importance (i.e., have a higher degree) in the multi-*omic* map versus the TR (*climbers*, see Methods and Figure 2A) pertain mostly to the MAPK Signaling Pathway (Figure 2B and Data Sheet 2, Table S19). This category is also highly enriched for *accomplished* nodes, thus validating the importance of this pathway in the disease. However, *climbers*, all representing genes shared between TR and EI, include molecules known to belong also to the GI interface (SFR, MAP2K4, MAP3K8), absent in the *accomplished*, implying the importance of the involvement of the host-microbiome interface, not taken into account in the TR map. In particular, Interleukin-1 Receptor Associated Kinase-4 (IRAK4, *climber*) is known to play a critical role in initiating response to foreign pathogens (Hofman and Vouret-Craviari, 2012) and was recently presented to the American College of Rheumatology (ACR), based on promising results on the control of B-cell-like diffuse large B-cell lymphoma (DLBCL), as a potential treatment for RA (Chaudahry and Al, 2012). In the network perspective, this choice calls for words of cautions. Indeed, while correlating with regression of some aspects of the disease, the control on IRAK4 affects the response to pathogens, and in particular IRAK4 inhibitors impacts on pDCs in RA patients (Chiang et al., 2011), therefore limiting the appropriate and immediate innate host response in case of bacterial infections (Figure 2C).

## Conclusion

The aim of the designed framework is to draw hypotheses that can support basic research and further clinical practice. In particular, we here highlight two major areas of application: support in the identification of novel drug targets (exemplified by GRB2); support in the identification of potential contraindication to novel therapies, i.e., support in the design of robust clinical trials (exemplified by IRAK4-inhibitors). While the former application joins other efforts in different clinical areas [such as on diabetes (Liu et al., 2007; Santiago and Potashkin, 2013), in cancer (Hwang et al., 2013), and on glioblastoma (Junhua et al., 2012)], the latter descends from the inclusion of numerous data types, including for the first time to our knowledge, the GI microbiome interface. The results discussed in this article are the output of the knowledge distilled from ~4000 selected molecules and ~15 public databases, a humongous amount of information carefully and often redundantly peer-reviewed by the scientific community. Future and ongoing research and the resulting discoveries will impact on the breadth and possibly on the topology of our map. To take into account these expected (and desirable) events, our map was drawn using open source programs and pathway molecules' standards to allow full map usability, editing and updating by the whole scientific community.

## Author contribution

Paolo Tieri built and analyzed the map; XiaoYuan Zhou performed pharmacological simulation; XiaoYuan Zhou and Lisha Zhu run functional and comparative analyses; Christine Nardini analyzed the connection to the GI microbiome; Paolo Tieri and Christine Nardini designed the study, analyzed the results and wrote the manuscript; XiaoYuan Zhou and Lisha Zhu contributed to write and revise the manuscript.

## Data sharing statement

All data are available publicly, our map is publicly available here: http://www.picb.ac.cn/ClinicalGenomicNTW/RAmultiomic.html

### Conflict of interest statement

The authors declare that the research was conducted in the absence of any commercial or financial relationships that could be construed as a potential conflict of interest.
