# Supplementary material for: Multi-omic landscape of rheumatoid arthritis: re-evaluation of drug adverse effects
Source: Front Cell Dev Biol. 2014 Nov 4;2:59. doi: 10.3389/fcell.2014.00059 (PMC4220167; doi:10.3389/fcell.2014.00059)
Supplement: Supplementary file 1 [file Presentation1.PDF]

## SUPPLEMENTARY MATERIAL

**Figure S1** Core interactome main connected component, high-resolution figure, zoomable down to the node name resolution
**Figure S2** Extended interactome main connected component, high-resolution figure, zoomable down to the node name resolution
**Tables S1-S14** method and datasets details, and molecules' lists
**Tables S15-S20** Topological and functional analysis results
**Interactomes** CI_EI.cys, Cytoscape-format Core and Extended interactome networks
