# Supplementary figures and images for: Multi-omic landscape of rheumatoid arthritis: re-evaluation of drug adverse effects
Source: Front Cell Dev Biol. 2014 Nov 4;2:59. doi: 10.3389/fcell.2014.00059 (PMC4220167; doi:10.3389/fcell.2014.00059)

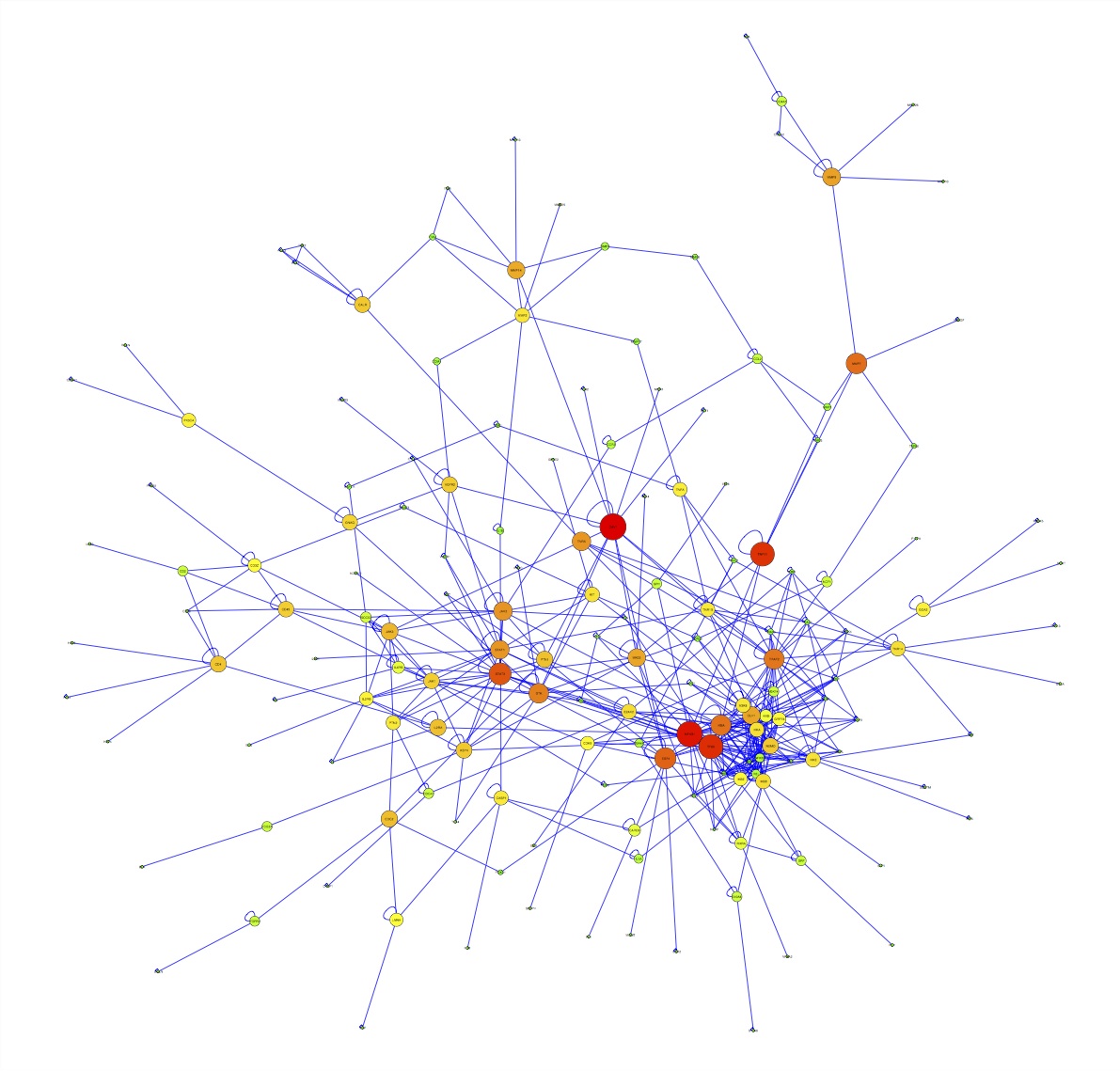

Supplement: Supplementary file 5 [file Image1.JPEG]

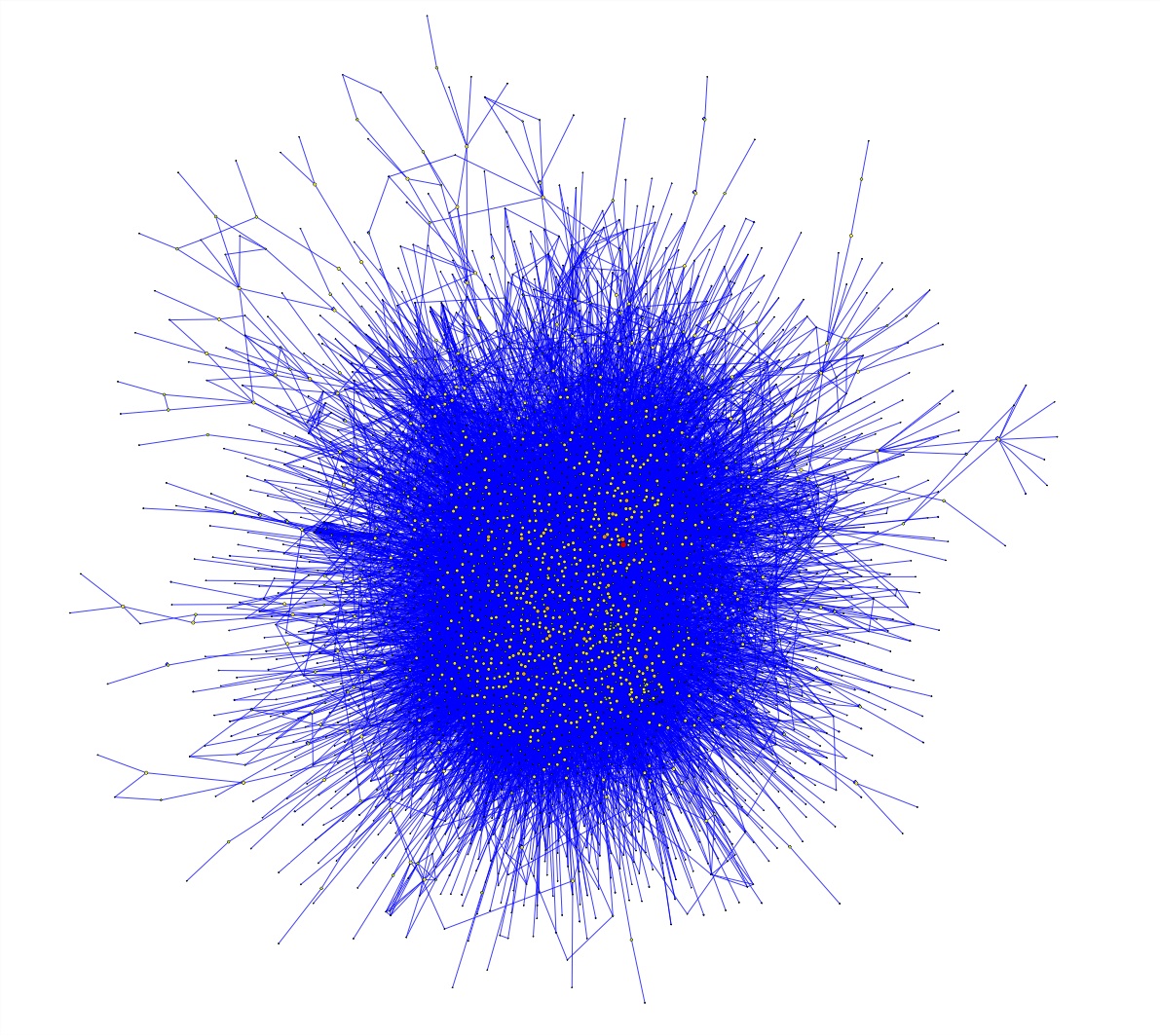

Supplement: Supplementary file 6 [file Image2.JPEG]
